# Treatment of Hypochondriasis in Two Schizophrenia Patients Using Clozapine

**DOI:** 10.1155/2017/5064047

**Published:** 2017-05-31

**Authors:** Antonio Tundo, Luca Proietti, Rocco de Filippis

**Affiliations:** Istituto di Psicopatologia, Via Girolamo da Carpi 1, 00196 Rome, Italy

## Abstract

Hypochondriasis (HYPO), an obsessive-compulsive spectrum disorder, is frequent in patients with schizophrenia (SCH) (20%), especially among those treated with clozapine (36.7%). Treatment options for OCS/OCD in patients under clozapine (CLZ) include combining clozapine with amisulpride/aripiprazole or a mood stabilizer, augmenting clozapine with a serotoninergic reuptake inhibitor, adding cognitive behavioural therapy, and gradually reducing dosage. No treatments have been proposed for HYPO in patients using clozapine so we examine these options in 2 cases and report the results. Among treatments delivered, only dosage reduction adequately worked. We recommend caution when thinking about escalating treatment and suggest trying it only when alternative interventions were not successful and weighing risk and benefits of this therapeutic strategy. Further research is needed to confirm the hypothesis that CLZ treatment induces hypochondriac symptoms, to investigate the prevalence of the phenomenon, and, mostly, to identify possible treatment strategies.

## 1. Introduction

Clinical evidence shows that patients with schizophrenia (SCH) have a higher prevalence of OCS (30%) and OCD (12%) than the general population (2-3%) [1, 2] and that in more than 70% of the cases the onset or aggravation of OCS takes place after the beginning of treatment with a second-generation antipsychotic (SGA), mainly clozapine (CLZ) (for a review see [3]).

The antiserotonergic properties (mostly the antagonism at 5-HT1C, 5-HT2A, and 5-HT2C receptors) and the putative proglutamatergic effect of CLZ may account for this observation (for a review see [3]). However, hypothesized causal interactions need further confirmation and an interaction between genetic/biological predispositions, psychosocial factors, and treatments could better explain the phenomenon [3].

A recent study showed that also hypochondriasis (HYPO) is more frequent in patients with SCH (20%) than in the general population (1%) and that its prevalence is higher in patients treated with CLZ (36.7%) than in those treated with other antipsychotics (6.7%) [4].

HYPO is an obsessive-compulsive spectrum disorder [5], so we can assume that the same pathogenetic hypothesis proposed for CLZ-induced OCS/OCD is implicated in CLZ-induced HYPO.

To our knowledge, no treatment guidelines are available for hypochondriasis symptoms in SCH patients treated with CLZ, so in 2 cases we tried the treatments proposed for OCS/OCD co-occurring with SCH in patients taking CLZ and reported the results. These treatments include combining clozapine with amisulpride/aripiprazole or a mood stabilizer, augmenting clozapine with a serotoninergic reuptake inhibitor (SRI), adding cognitive behavioural therapy (CBT), and gradually reducing dosage (for a review see [6–9]). We assessed psychopathological and clinical conditions as well as general functioning using the Positive and Negative Symptom Scale total score [10], Clinical Global Impression-Severity [11], Global Assessment of Functioning Scale [12], and, after HYPO onset, Hypochondriasis Y-BOCS Scale [13]. The scales were administered at each visit, every month during the acute phase, and every 3-4 months during the maintenance phase.

## 2. Case Reports

### 2.1. Case Report A

Mr. F. is a 52-year-old man, single, and employed. He had no a personal or familiar history of OCD or HYPO. At 31 years, he developed schizo-affective disorder (SA) (DSM-IV criteria), successfully treated (Table 1) with clozapine (200 mg/day), lithium salts (serum level 0.60 mEq/l), and valproate (600 mg/day, serum level 50 *μ*gr/ml). After 5 years of remission, we discontinued valproate and reduced clozapine to 150 mg/day. When F. was 47 years old, he developed severe HYPO (DSM-IV criteria) (Table 2) with headache, dizziness, fear of having a neurological disease, and reassurance seeking (frequent neurological visits and calls). We treated unsuccessfully HYPO: (a) adding sertraline (150 mg/day) and discontinuing it after 3 months for psychotic symptoms reactivation (the Positive and Negative Symptom Scale total score increased from 44 to 72); (b) adding valproate (900 mg/day, serum level 78 *μ*gr/ml) (4 months); (c) adding aripiprazole (20 mg/day) (2 months). The combination of pharmacotherapy and CBT (37 sessions) partially improved HYPO. Lastly, we reduced the clozapine dosage from 150 to 75 mg/day (25 mg for month) and HYPO gradually remitted (Table 2). HYPO remission, as well as SA remission, continued for 3 years.

### 2.2. Case Report B

Mr. B. is a 48-year-old man, single, and employed. He had a personal history of panic attacks but not a personal or familiar history of OCD or HYPO. When he was 19 years old, he developed SCH successfully treated with clozapine (500 mg/day) in combination with valproate (1000 mg/day, serum level 86 *μ*gr/ml) (Table 1). Seven years ago, we discontinued valproate and reduced clozapine (400 mg/day). When B. was 45 years old, he developed a very severe HYPO disorder (DSM-IV criteria) (Table 2) with dyspnea, fear of having a cardiac disease, and reassurance seeking (frequent medical visits and hospital admissions). B. refused CBT and we treated HYPO unsuccessfully: (a) adding paroxetine (30 mg/day), sertraline (150 mg/day), and clomipramine (50 mg/day) (resp., 2, 3, and 2 months); (b) adding valproate (1500 mg/day, serum level 94 *μ*gr/ml) (3 months); (c) adding valproate (1500 mg/day, serum level 91 *μ*gr/ml) plus amisulpride (600 mg/day) (4 months). Lastly, we reduced clozapine dosage (100 mg/die) without changing valproate and amisulpride doses and HYPO dramatically improved (Table 1). HYPO improvement, as well as SCH remission, continued for 2 years.

## 3. Discussion

To our knowledge, this is the first report on treatment of HYPO secondary to CLZ use. Among the attempted therapeutic strategies, combining clozapine with amisulpride/aripiprazole or valproate did not work, augmentation with a SRI did not work (1 case) or worsened psychosis (1 case), and CBT partially worked (1 case). Only dosage reduction adequately worked.

Although the main focus of this report was evaluating the possible treatment strategies for hypochondriasis symptoms co-occurring with SCH in patients using CLZ, it suggests that, as previously reported for OCS [14], hypochondriasis is a dose and treatment duration dependent side effect of CLZ.

Undoubtedly, CLZ is the more effective antipsychotic for treatment-resistant psychoses and has important protective effects against suicidal behaviour resulting in lowest mortality of patients with SCH [15–17]. Therefore, clinicians should consider that CLZ could induce the onset or aggravation not only of OCS/OCD, as reported in some studies, but also of HYPO and that this disorder needs a specific treatment. In this regard the dosage reduction, if possible, proved to be a useful option. We recommend caution when thinking about escalating treatment and suggest trying it only when alternative interventions miscarried and weighing risk and benefits of this therapeutic strategy. The control of psychotic symptoms must remain a priority in the treatment of patients with SCH.

Further research is warranted to confirm the hypothesis that CLZ treatment induces hypochondriac symptoms, to investigate the prevalence of the phenomenon, and, mostly, to identify possible treatment strategies.

## Figures and Tables

**Table 1:** Psychopathological, clinical, and functioning assessments before clozapine introduction and after clozapine response.

Scale	Case A		Case B	
	Before clozapine introduction	After clozapine response	Before clozapine introduction	After clozapine response
Positive and Negative Symptom Scale total score [10]	86	43	98	50
Global Assessment of Functioning Scale [11]	45	75	40	63
Clinical Global Impression-Severity [12]	4	1	5	2

**Table 2:** Hypochondriasis Y-BOCS scores before and after clozapine dosage reduction.

Item	Case A		Case B	
	Before clozapine dosage reduction	After clozapine dosage reduction	Before clozapine dosage reduction	After clozapine dosage reduction
Time occupied by hypocondriac thoughts	3	1	4	1
Interference of hypocondriac thoughts with functioning	3	0	4	2
Distress caused by hypocondriac thoughts	3	1	4	2
Resistance against hypocondriac thoughts	3	2	4	2
Degree of control over hypochondriac thoughts	3	1	4	2
Insight into hypochondriacal thoughts	3	1	4	2
Time spent performing hypochondriac behaviors	2	1	4	1
Interference due to hypochondriac behaviors	2	0	4	1
Distress associated with hypochondriac behaviors	3	1	4	1

*Total*	*25*	*8*	*36*	*13*

%* reduction*		*68*		*64*
